# Spatial Disassociation of Disrupted Functional Connectivity for the Default Mode Network in Patients with End-Stage Renal Disease

**DOI:** 10.1371/journal.pone.0161392

**Published:** 2016-08-25

**Authors:** Xiaofen Ma, Junzhang Tian, Zhanhong Wu, Xiaopeng Zong, Jianwei Dong, Wenfeng Zhan, Yikai Xu, Zibo Li, Guihua Jiang

**Affiliations:** 1 Department of Medical Imaging, Guangdong Provincial No.2 People’s Hospital, Guangzhou City, Guangdong province, PR China; 2 Biomedical Research Imaging Center and Department of Radiology, University of North Carolina at Chapel Hill, Chapel Hill, North Carolina, United States of America; 3 Department of Mathematics, Guangdong Pharmaceutical University, Guangzhou City, Guangdong province, PR China; 4 Department of Medical Imaging Center, Nanfang Hospital, Southern Medical University, Guangzhou City, Guangdong province, PR China; University of Texas at Austin, UNITED STATES

## Abstract

**Purpose:**

To investigate the aberrant functional connectivity of the default mode network (DMN) in patients with end-stage renal disease (ESRD) and their clinical relevance.

**Materials and Methods:**

Resting-state functional MRI data were collected from 31 patients with ESRD (24 men, 24–61 years) and 31 age- and gender-matched healthy controls (HCs, 21 men, 26-61years). A whole-brain seed-based functional connectivity analysis of these collected R-fMRI data was performed by locating the seeds in the posterior cingulate cortex (PCC) and ventromedial prefrontal cortex (vmPFC) to investigate the functional connectivity of the posterior and anterior DMN over the whole brain, respectively.

**Results:**

Compared to the HCs, the patients exhibited significantly decreased functional connectivity with the PCC in the left middle temporal gyrus, the right anterior cingulate gyrus, and the bilateral medial superior frontal gyrus. For the vmPFC seed, only the right thalamus showed significantly decreased functional connectivity in the patients with ESRD compared to HCs. Interestingly, functional connectivity between the PCC and right medial superior frontal gyrus exhibited a significantly positive correlation with the hemoglobin level in the patients.

**Conclusion:**

Our findings suggest a spatially specific disruption of functional connectivity in the DMN in patients with ESRD, thereby providing novel insights into our understanding of the neurophysiology mechanism that underlies the disease.

## Introduction

End-stage renal disease (ESRD) is a disease characterized by multi-organ dysfunction, which typically occurs when chronic renal failure progresses to a point where the kidneys are permanently functioning at less than 10% of their capacity [1]. ESRD is not only accompanied with central nervous system abnormalities (e.g., white matter lesions, cerebral atrophy and myelinoclasis) [2], but also results in various neurological problems (e.g., Wernicke’s encephalopathy, uremic encephalopathy and dialysis encephalopathy) [3]. Moreover, ESRD significantly elevates the risk for developing cognitive impairments [4–6].

Recently, neuroimaging techniques have been used to investigate the neural mechanisms underlying ESRD-related neurological complications. For example, conventional MR imaging studies have shown that focal white matter lesions are more common in ESRD patients (56%) than in the normal population (27%) [7,8]. Based on single-photon emission computed tomography, arterial spin-labeling MR perfusion imaging and magnetic resonance spectroscopy, ESRD-related changes in cerebral metabolism and function have also been documented [9–11]. Further evidence from diffusion tensor imaging studies indicates that ESRD is associated with widespread disruptions of white matter integrity [12–14]. These studies collectively indicate the disorganized brain architecture induced by ESRD.

More recently, several groups have begun to apply resting-state functional magnetic resonance imaging (R-fMRI), a promising technique to depict intrinsic functional connectivity networks [15–17] to studies of ESRD [18–21]. With this technique, a consistent finding is disrupted functional integration of the default mode network (DMN) in ESRD [18,19,21,22], based on methods of regional homogeneity, independent component analysis or nodal centrality. It is well documented that the DMN is functionally heterogeneous with different connectivity profiles between its anterior and posterior parts [23–25]. However, whether and how the anterior and posterior components of the DMN are differentially involved in ESRD are largely unknown.

In the current study, we performed a whole-brain seed-based functional connectivity analysis of R-fMRI data collected from 31 neurologically asymptomatic patients with ESRD to investigate their functional connectivity maps of the DMN regions over the whole brain. Specifically, the seeds were centered at the posterior cingulate cortex (PCC) and ventromedial prefrontal cortex (vmPFC), respectively, to examine spatial specificity of ESRD-related alterations. Finally, ESRD-related functional connectivity alterations were correlated with neuropsychological tests and biomechanical variables of the patients.

## Materials and Methods

### Participants

This study was approved by the Research Ethics Review Board of Guangdong Provincial No.2 People’s Hospital, and written informed consent was obtained from each participant. A total of thirty-four patients with ESRD (all right-handed) were enrolled in this study from the renal transplantation department of our hospital between August 2011 and July 2013. Exclusion criteria included: (1) psychiatric disorders or major neurologic disorders (e.g., severe head injury, stroke, epilepsy, dementia, anxiety, depression or visible lesions) according to an experienced physician (G. X., with 20-year experience in neurology); (2) ischemic diseases including acute ischemic cerebrovascular disease, acute peripheral arterial occlusion, advanced liver or heart failure; (3) asymptomatic coronary ischemia by electrocardiogram testing; (4) a history of diabetes; and (5) substance abuse including drugs, alcohol or cigarettes. Conventional MR images were examined by an experienced radiologist (W. L., with 20 years of experience in neuropathology), who was blinded to whether the images were from the patient or control group. Three patients were excluded due to abnormal hyper-intensities in the T2-FLAIR MR images [3]. Therefore, the final study population included 31 patients with ESRD (24 men and 7 female, mean age 39.9 ± 9.6 years, range 24–61 years).

Thirty-one age- and gender-matched HCs (all right-handed; 32 males; mean age 41.5 ± 10.6 years, range 22–58 years) were recruited from the local community. All the HCs had no physical diseases or history of psychiatric or neurologic diseases.

All the participants underwent a neuropsychological test involving the mini-mental state examination (MMSE) [26], and the evaluation of systolic and diastolic blood pressure. ESRD group completed multiple biochemical tests after the hemodialysis (within 36 hours) but before the MR imaging (within 24 hours). The biochemical tests included Scr (serum creatinine), BUN (blood urea nitrogenurea), cholesterol, hemoglobin, serum kalium and serum calcium. Out of the 31 patients with ESRD, 20 (64.5%) had hypertension, and 6 (19.3%) had hyperlipidemia. In the current study, the patients with anemia were treated with ferrous succinate or polysaccharide iron complex.

All the demographic and clinical data are summarized in Table 1.

**Table 1:** Demographics and clinical characteristics of all participants.

	ESRD (n = 31)	HCs (n = 31)	P-value
Gender (M/F)	24/7	21/10	0.393^a^
Age (years)	39.9 ± 9.6 (24–61)	42.7 ± 8.5 (26–61)	0.216^b^
Education (years)	11.8 ± 3.3 (3–16)	10.8 ± 2.8 (6–16)	0.174^b^
MMSE	29.1 ± 0.8 (28–30)	29.8 ± 0.5 (28–30)	<0.001^b^
Systolic blood pressure	172.9 ± 9.6 (160–180)	100.9 ± 7.2 (90–115)	<0.001^b^
Diastolic blood pressure	95.4 ± 6.1 (85–100)	70.7 ± 5.9 (60–80)	<0.001^b^
Dialysis duration (months)	16.0 ± 6.6 (6–30)		
Serum calcium (mmol/L)	2.3 ± 0.2 (1.9–2.9)		
Serum kalium (mmol/L)^c^	4.6 ± 0.8 (3.0–6.3)		
Hemoglobin (g/L)^d^	104.0 ± 24.7 (56–158)		
Serum creatinine (μmol/L)	783.3 ± 402.2 (86–1458)		
Blood urea nitrogenurea (mmol/L)	17.7 ± 8.1 (4.1–30.3)		

Values are represented as mean ± SD (min—max). ESRD, end-stage renal disease; HCs, healthy controls; M, male; F, female; MMSE, the Mini-Mental Status Examination.

^a^The P-value was obtained by chi-square test.

^b^The P-values were obtained by two-side two-sample t tests.

^c^Data were missed for two patients.

^d^Data were missed for three patients.

### Image acquisition

All participants were scanned on a 1.5-T MR scanner (Achieva Nova-Dual, Philips, Best, the Netherlands) in the Department of Medical Imaging center at Guangdong No. 2 Provincial People’s Hospital. None of the subjects were taking any medications at the time of the scans. The conventional imaging sequences, including T1-weighted images and T2-FLAIR images, were obtained for each participant to detect clinically silent lesions. During the R-fMRI data scanning, the participants were asked to lie quietly with their eyes closed and to not think of anything specific while in the scanner. The scan lasted 8 minutes, and 160 volumes were obtained for each participant. The R-fMRI acquisition parameters were as follows: 33 axial slices; repetition time (TR) = 3,000 ms; echo time (TE) = 50 ms; flip angle = 90°; slice thickness = 4.5 mm; no gap; matrix = 128 × 128 and field of view (FOV) = 230 × 230 mm^2^. After scanning, all the participants were asked questions to verify the degree of their cooperation. Additionally, individual high-resolution anatomical images were also acquired using a T1-weighted three-dimensional volumetric magnetization-prepared rapidly acquired gradient-echo sequence: 160 axial slices; TR = 25 ms; TE = 4.1 ms; FA = 30°; slice thickness = 1.0 mm; no gap; matrix = 256 × 256; and FOV = 230 × 230 mm^2^.

### Image preprocessing

Data preprocessing was performed using the SPM12 package (http://www.fil.ion.ucl.ac.uk/spm/software/spm12/) and GRETNA package [27], including i) removal of the first five volumes to allow T1 equilibration effects; ii) realignment to correct for spatial displacements due to head motion; iii) co-registration to structural images; iv) spatial normalization into the Montreal Neurological Institute space by applying deformation filed derived from tissue segmentation of structural images; v) spatial smoothing (Gaussian kernel of 6-mm full width at half maximum); vi) removal of linear trend; vii) temporal band-pass filtering (0.01–0.1 Hz); and viii) regression of several nuisance signals of white matter signal, cerebrospinal fluid signal and head-motion profiles.

Recent studies have highlighted residual head-motion effects on intrinsic functional connectivity [15,28]. In the current study, we first excluded participants with head motion > 3 mm of displacement or > 3 degree of rotation in any direction. Then, we examined both gross (i.e., maximum and root mean square) and micro (mean frame-wise displacement) head-motion summary measures and found no significant between-group differences (all *P*s > 0.05). Furthermore, we employed a 24-parameter instead of 6-parameter head-motion model during the regression of nuisance signals [29], an efficiency strategy to control for head motion effects [30]. Finally, we treated all the summary head-motion measures as covariates at the group-level comparisons [31]. After these efforts, we believe that head-motion effects were mitigated as much as possible for the current data. Of note, how to attenuate head-motion effects is an ongoing topic of research, and there are other alternative strategies to deal with this issue [32,33].

### Seed-based functional connectivity

Although typically regarded as a homogenous network, the DMN is functionally heterogeneous, with particularly striking differences in connectivity patterns between the anterior and posterior DMN [23]. Thus, in the current study, we studied functional connectivity of both anterior and posterior DMN by locating the seeds in the PCC (Talairach coordinates = [–2–51 27]) and vmPFC (Talairach coordinates [2 54–3]), respectively [23]. Their counterparts in the MNI space converted according to [34] were then used as centers to generate two spherical regions of interest (ROIs) with radius = 6 mm. For each participant, a reference time series was then obtained for each ROI by averaging all the voxels’ time series within it. The resulting reference time series were further correlated with the time series over the entire brain in a voxel-wise manner, thereby generating two functional connectivity maps individually. Finally, a Fisher’s r-to-z transformation was applied to the resulting whole-brain correlation maps to improve the normality of the correlation coefficients.

### Statistical analysis

#### Functional connectivity map and between-group difference

For each group, a random-effect one-sample t-test was performed in a voxel-wise manner to determine regions that showed functional connectivity with the seeds (i.e., PCC and vmPFC). A random field theory as performed in the SPM toolbox was used to account for the multiple comparison issue (*P* < 0.05, corrected, cluster size > 10 voxels). To further identify regions whose functional connectivity with the seeds differed between the ESRD and HC groups, a voxel-wise multiple general linear model was implemented with age, gender, education and summary head-motion measures as covariates. To correct for the multiple comparison issue, the Alpha-Sim procedure [35] was implemented in the REST by combining the height threshold of *P* < 0.001 [36] and extent threshold of *P* < 0.05, which corresponded to a corrected *P* < 0.05. All the results were mapped onto the cortical surfaces for visualization using the BrainNet Viewer package [37].

#### Brain-behavior relationship

For each region showing significantly different functional connectivity between the ESRD and HC controls, the Pearson correlation coefficient was calculated to assess the relationship between mean function connectivity strength of the region (after Fisher’s r-to-z transformation) and clinical variables (dialysis duration, calcium level, kalium level, hemoglobin level, creatinine level and urea level) in the patient group.

## Results

### Demographic and clinical characteristics

The demographic, biochemical and clinical characteristics for all the participants are shown in Table 1. There were no significant differences in gender (*P* = 0.393), age (*P* = 0.216) or education level (*P* = 0.174) between the ESRD and HC groups. Compared with the HCs, the ESRD patients had significantly higher systolic and diastolic blood pressure values and lower MMSE scores (*P* < 0.001). The mean duration of hemodialysis for the patient group was 16.0 ± 6.6 months. The mean calcium, kalium, hemoglobin, creatinine, and urea levels for the patients were 2.3 ± 0.2 mmol/L, 4.6 ± 0.8 mmol/L, 104.0 ± 24.7g/L, 783.3 ± 402.2 μmol/L and 17.7 ± 8.1 mmol/L, respectively. Notably, the serum calcium levels were corrected with serum albumin levels using the Payne's formula [38].

### Functional connectivity maps

**Fig 1:**
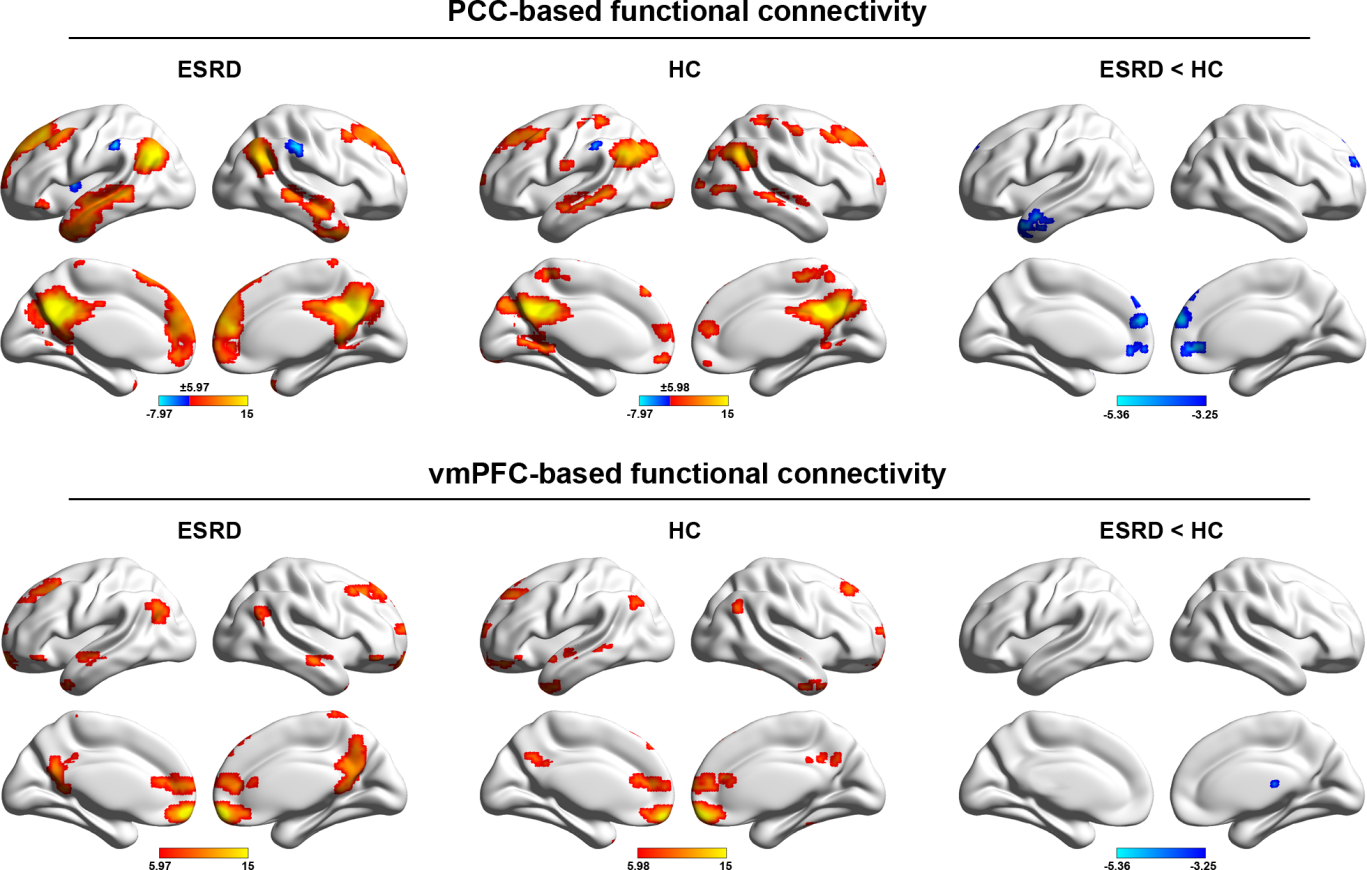
Within-group DMN patterns and between-group differences. The color bars represent the T scores. The results were mapped onto the brain surface using the BrainNet viewer software.

Fig 1 shows the DMN functional connectivity patterns for the ESRD and HC groups and between-group differences. Overall, our PCC-based and vmPFC-based functional connectivity analyses largely replicated numerous previous studies of the DMN topography that predominantly embraced the medial prefrontal cortex, dorsolateral prefrontal cortex, lateral temporal cortex, and post-medial parietal cortex. We also note that PCC-based functional connectivity analysis revealed a spatially more extensive DMN pattern relative to the vmPFC.

Compared with the HC, four clusters were found to show significantly decreased functional connectivity with the PCC in the patients with ESRD, involving the left middle temporal gyrus, the right anterior cingulate gyrus, and the bilateral medial superior frontal gyri. For the vmPFC seed, only the right thalamus showed significantly decreased functional connectivity in patients with ESRD compared to HCs (Fig 1).

### Relationship between DMN functional connectivity and clinical variables

**Fig 2:**
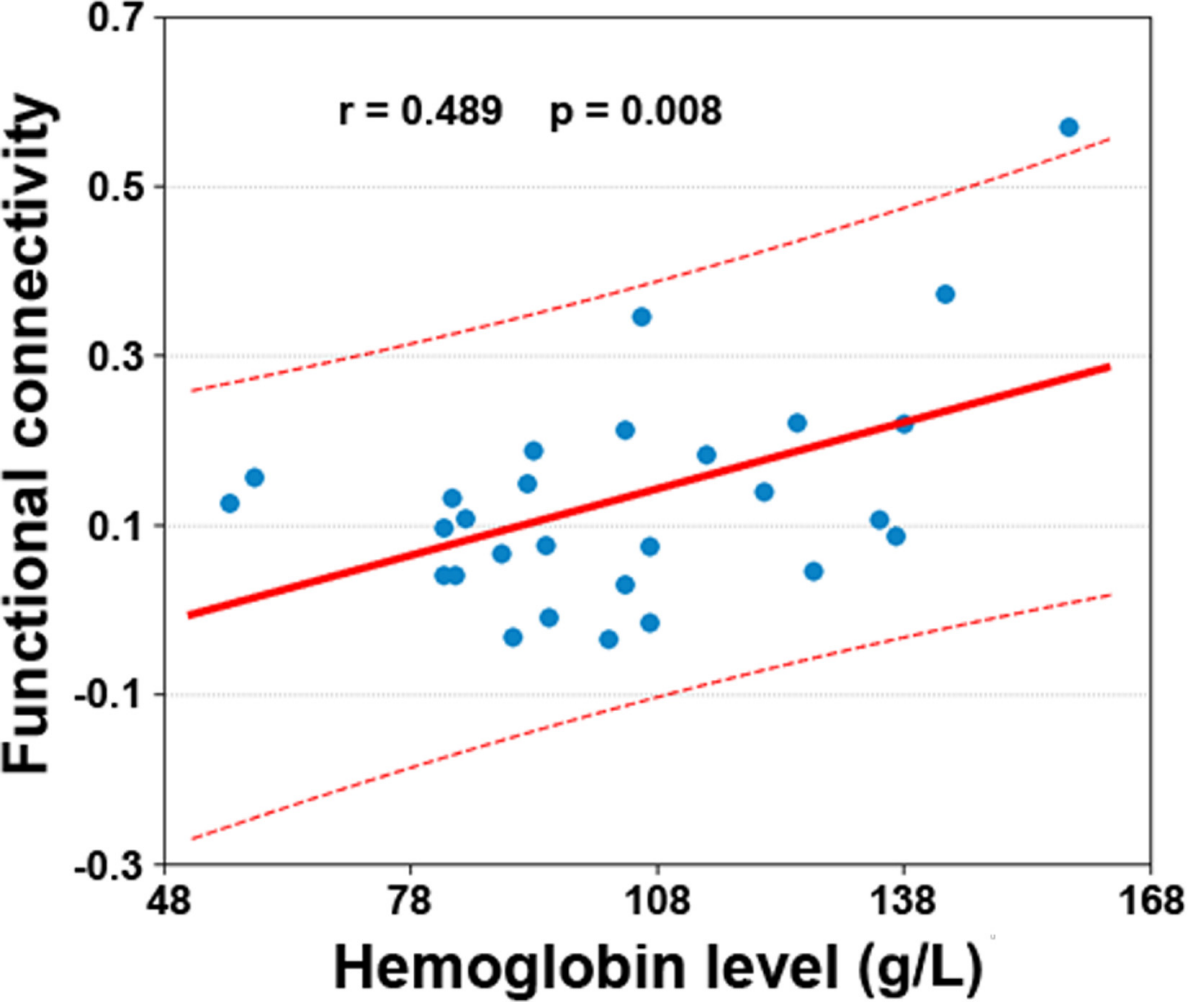
Scatter plot of the relationship between the PCC-right medial superior frontal gyrus functional connectivity and hemoglobin levels in the patients.

Functional connectivity between the PCC and right medial superior frontal gyrus exhibited a significantly positive correlation with the hemoglobin level (r = 0.489, *P* = 0.008) (Fig 2). We did not correlate ESRD-related functional connectivity alterations with the MMSE scores in the patients due to the narrow range distribution of the values (28–30).

### Reproducibility analyses

**Fig 3:**
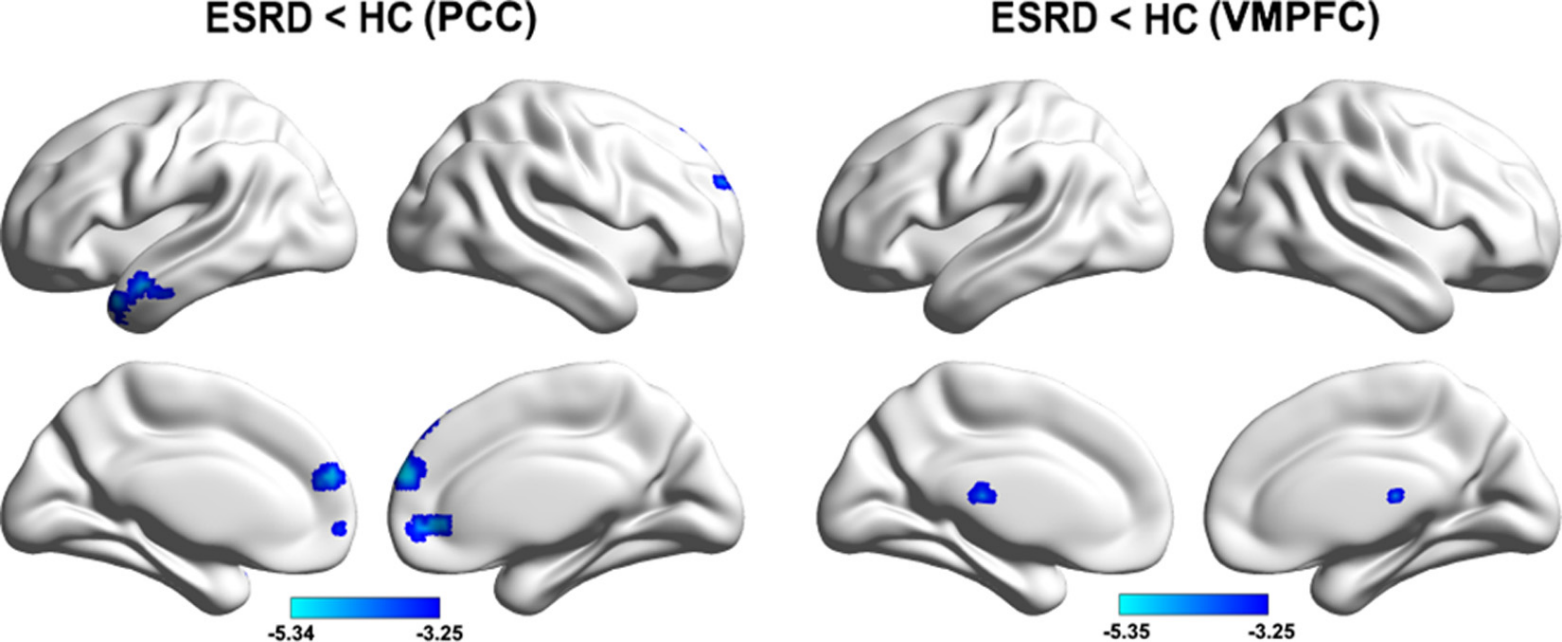
Between-group functional connectivity differences after excluding two patients with anemia.

In the current study, we noted that two patients had very low hemoglobin values (56 g/L and 59 g/L), suggesting severe anemia. Therefore, we reanalyzed our data after excluding these two patients to test the extent to which our main findings were affected. We found that the results were largely preserved for both whole-brain functional connectivity differences (Fig 3) and MRI-clinical correlations (r = 0.590, *P* = 0.002). In addition, for the ESRD patients included in the current study, 20 (64.5%) had hypertension and 6 (19.3%) had hyperlipidemia. Thus, we further compared the functional connectivity between the ESRD patients with hypertension/hyperlipidemia and those without hypertension/hyperlipidemia for each cluster that exhibited functional connectivity differences between the ESRD and HC groups. No significant differences were found for any cluster (*P* > 0.05). Furthermore, we performed a voxel-wise comparison of whole-brain functional connectivity between the ESRD patients with hypertension/hyperlipidemia and those without hypertension/ hyperlipidemia. Again, no significant differences were observed (*P* > 0.05, corrected with the Alpha-Sim procedure). All these reproducibility analyses indicate little effects of anemia, hypertension and hyperlipidemia on the reported results.

## Discussion

This study employed R-fMRI to evaluate the DMN functional connectivity in patients with ESRD. Although typically regarded as a homogenous network, accumulating evidence indicates functional heterogeneity within the DMN. Specifically, evidence from task-based activation studies shows that the anterior vmPFC and posterior PCC, two key nodes within the DMN, act independently across a wide array of cognitive tasks. Moreover, functional connectivity studies from both resting state and cognitive tasks reveal different connectivity patters between the vmPFC and PCC [23–25]. The anterior-posterior functional dissociation within the DMN implies differences in their interactions with other networks and highlights the need for researchers to treat them individually. In response, we examined both anterior and posterior DMN functional connectivity in ESRD by seeding the ROIs in the PCC and vmPFC, respectively. We found that patients with ESRD mainly exhibited decreased within-DMN functional connectivity when the seed was located in the PCC while decreased frontal-subcortical functional connectivity was determined when the seed was located in the vmPFC. These findings provide new insights into our understanding of neural abnormalities in ESRD.

We found that compared with HCs, patients with ESRD showed decreased functional connectivity in the anterior cingulate cortex, middle temporal gyrus and medial superior frontal cortex when the seed was located in the PCC. These structures are typical DMN components, suggesting decreased within-DMN functional integration in ESRD. This is consistent with previous R-fMRI studies based on regional homogeneity [18] and independent component analysis methods [19] in ESRD. Using MR spectroscopy [11,39], diffusion-tensor imaging [14], and voxel-based morphometry [10], many previous studies have demonstrated ESRD-related abnormalities in brain biochemistry and structure of multiple DMN regions. These abnormalities may be the possible biochemical and/or structural basis for the disrupted DMN functional connectivity observed here. Functionally, the DMN is engaged in a broad array of cognitive processing related to self-awareness, episodic memory, and interactive modulation between the internal brain activities and external tasks [40,41]. Before any overt neurological manifestation, patients with ESRD often develop various cognitive deficits involving attention, processing speed [42], executive function [4], and memory [43]. Thus, we speculated that the decreased within-DMN functional integration might in part underlie these cognitive disturbances in ESRD. Future follow-up studies are required to provide more complete neuropsychological test of ESRD to find the real relation between the neuropsychological test in ESRD patients and DMN.

Interestingly, we found a positive correlation between PCC-medial superior frontal gyrus function connectivity and the hemoglobin levels in patients with ESRD. Previous studies have shown that long-term hemodialysis could lead to cerebral abnormalities of oxygenation [10] and cerebral blood flow in ESRD [44–46], which could significantly affect the brain function and cerebral circulation [47–50]. Recent studies have highlighted important roles of cerebral blood flow and metabolism in establishing and retaining interregional functional coordination in the brain [51,52]. Given previous findings that low hemoglobin is associated with poor mental health in ESRD [46,53,54], further insights into this issue could benefit from simultaneously recording MR spectroscopy, ASL perfusion, R-fMRI, and neuropsychological data of the same cohort of patients in future.

When seeded in the vmPFC, patients with ESRD were found to show decreased functional connectivity with the thalamus. vmPFC is a key structure implicated in emotional and cognitive processing by interacting with a number of subcortical structures, including the thalamus [23,55,56]. Therefore, disruption of both structural and functional connectivity between the thalamus and frontal DMN regions are frequently reported for depressive individuals [57,58]. Patients with ESRD typically present with mood-related problems, such as adaptive behavior of fear. Previous studies have shown that the paraventricular nucleus of the thalamus (PVT), one putative stress sensor [59,60], constitutes a novel circuit essential for establishment of fear memory, the expression of fear responses and adaptive behavior of fear [61]. Moreover, ESRD patients are prone to develop depression [62]. Thus, we deduce that the decreased functional connectivity between the vmPFC and the thalamus observed here may account for neurocognitive dysfunctions and in particular mood-related processing (e.g., adaptive behavior of fear), which ultimately contribute to the susceptibility of ESRD patients to develop depression. If this deduction holds true, an interesting future topic is to test whether cognitive training that can enhance the thalamic and DMN functional connectivity may improve cognitive function in patients with ESRD. However, there is another possibility that the current patients may be complicated by depression, which further leads to the observed functional connectivity decrease between the vmPFC and thalamus. Future studies are needed to clarify this issue.

## Limitations

First, the sample size was relatively small and the MRI scanning parameters were suboptimal (e.g., 1.5T scanner and anisotropic voxels) for the current dataset. However, our previous studies based on this dataset consistently demonstrate ESRD-related functional connectivity disruptions of the DMN [21,22], which are largely comparable with studies utilizing optimal imaging parameters on 3T MRI scanners [18,19]. This implies the validity of the current dataset to study functional connectivity of the DMN in ESRD. Nevertheless, we want to emphasize that future studies with a large cohort of participants are needed to examine the reproducibility of our findings using more advanced techniques and optimized parameters. Second, due to the cross-sectional design of the current study, we cannot address how the DMN functional connectivity changes dynamically in response to the progression of chronic kidney disease (CKD). Thus, future longitudinal studies addressing DMN functional connectivity and changes in neuropsychological tests in CKD patients with different degrees of kidney function may provide further insight on the time course of alterations in brain functional connectivity and neuropsychological behavior as kidney function deteriorates. Third, consistent with a recent R-fMRI study [19], several comorbidities associated with ESRD (e.g., anemia, hypertension and hyperlipidemia) existed for the patients in the current study. Therefore, it is likely that the observed DMN function connectivity abnormalities are a common consequence of both ESRD and these comorbidities. Although our exploratory analyses revealed non-significant effects of these factors on our results, it may be due to the small sample size. Given the influences of these factors on BOLD signals [63,64], it is an interesting topic in the future to systematically investigate how these factors impact functional networks of the brain. Additionally, it should be noted that there are still other factors that may contribute to the current findings given the end stage of the disease, such as depression, a common complication in ESRD. However, the current samples did not undergo neuropsychological tests for these psychological disorders, thus our findings should be interpreted with caution. Finally, we did not collect clinical information such as hematocrit, mean hemodialysis session duration and prevalence of intradialytic hypotension for the patients, limiting us to examine how ESRD-related functional connectivity alterations are related to these specific clinical variables in the patients. Future studies can address these important issues by using more rigorous experimental design and stricter enrollment criteria. Finally, accumulating evidence suggests that the DMN functional connectivity reflects underlying structural pathways [59, 60]. Therefore, it would be interesting to study whether the disrupted DMN functional connectivity observed here has a structural substrate.

## Conclusion

The current study demonstrates the disconnectivity between the PCC and the anterior cingulate and middle temporal gyrus regions, the vmPFC and the thalamus in the brain of patients with ESRD. Moreover, the disconnectivity is related to biochemical parameters in these patients. These findings provide novel insights into the neurophysiological mechanism of asymptomatic patients with ESRD.
